# Supplementary material for: A high-resolution mRNA expression time course of embryonic development in zebrafish
Source: eLife. 2017 Nov 16;6:e30860. doi: 10.7554/eLife.30860 (PMC5690287; doi:10.7554/eLife.30860)
Supplement: Supplementary file 6. [file elife-30860-supp6.zip › biolayout-clusters-files/Cluster173-genes.html]

Cluster173


# Cluster173: Genes

| Ensembl ID | Gene Name | Chr | Start | End | Biotype |
| --- | --- | --- | --- | --- | --- |
| ENSDARG00000036678 | TRMT10A | 7 | 18452654 | 18460613 | protein\_coding |
| ENSDARG00000044627 | alg8 | 21 | 22855862 | 22887702 | protein\_coding |
| ENSDARG00000079625 | atr | 2 | 16715406 | 16780917 | protein\_coding |
| ENSDARG00000104765 | chek1 | 21 | 22521549 | 22528565 | protein\_coding |
| ENSDARG00000041223 | fam173a | 3 | 14984360 | 14994294 | protein\_coding |
| ENSDARG00000026224 | fanci | 25 | 8370497 | 8402103 | protein\_coding |
| ENSDARG00000001447 | mier1b | 2 | 5899600 | 5924234 | protein\_coding |
| ENSDARG00000054473 | tinf2 | 7 | 23752496 | 23768904 | protein\_coding |
